# The influence of pharmacist-led collaborative care on clinical outcomes in type 2 diabetes mellitus: a multicenter randomized control trial

**DOI:** 10.3389/fpubh.2024.1323102

**Published:** 2024-02-27

**Authors:** Muhammad Zahid Iqbal, Saad Saeed Alqahtani, Naeem Mubarak, Sara Shahid, Rafiuddin Mohammed, Abid Mustafa, Amer Hayat Khan, Muhammad Shahid Iqbal

**Affiliations:** ^1^Department of Pharmacy Practice, Faculty of Pharmaceutical Sciences, Lahore University of Biological and Applied Sciences, Lahore, Pakistan; ^2^Department of Clinical Pharmacy, College of Pharmacy, King Khalid University, Abha, Saudi Arabia; ^3^Department of Health Informatics, College of Health Sciences, Saudi Electronic University, Riyadh, Saudi Arabia; ^4^Department of Pharmaceutics, Faculty of Pharmaceutical Sciences, Lahore University of Biological and Applied Sciences, Lahore, Pakistan; ^5^Discipline of Clinical Pharmacy, School of Pharmaceutical Sciences, Universiti Sains Malaysia, Penang, Malaysia; ^6^Department of Clinical Pharmacy, College of Pharmacy, Prince Sattam Bin Abdulaziz University, Al-Kharj, Saudi Arabia

**Keywords:** diabetes mellitus-type 2, blood pressure, HBA1c, DMTAC clinics, lipid profile, intervention group, control group

## Abstract

**Background:**

Health care providers are mandated to deliver specialized care for the treatment and control of type 2 diabetes mellitus. In Malaysia, Diabetes Medication Therapy Adherence Clinics (DMTAC) in tertiary hospitals have designated pharmacists to administer these services.

**Objective:**

To assess the effects of pharmacist-led interventions within DMTAC on the outcomes of patients with type 2 diabetes mellitus in two distinct hospitals in Kedah, Malaysia.

**Methods:**

Patients with type 2 diabetes were randomly selected from the two hospitals included in this study. The study population was divided into two equal groups. The control group consisted of 200 patients receiving routine care from the hospitals. On the other hand, the intervention group included those patients with type 2 diabetes (200), who received separate counseling sessions from pharmacists in the DMTAC departments along with the usual treatment. The study lasted 1 year, during which both study groups participated in two distinct visits.

**Results:**

Parametric data were analyzed by a paired *t*-test and one-way ANOVA, while non-parametric data were analyzed by a Chi-squared test using SPSS v24. A *p* < 0.05 was considered statistically significant. The study presented the results of a greater reduction in HBA1c levels in the intervention group compared to the control group, i.e., 3.59 and 2.17% (*p* < 0.001). Moreover, the Systolic and Diastolic values of BP were also significantly reduced in the intervention group, i.e., 9.29 mmHg/7.58 mmHg (*p* < 0.005). Furthermore, cholesterol levels were significantly improved in patients in the intervention group, i.e., 0.87 mmol/L (*p* < 0.001).

**Conclusion:**

Based on the findings of the current study it has been proven that the involvement of pharmacists leads to improved control of diabetes mellitus. Therefore, it is recommended that the government initiate DMTAC services in both private and government hospitals and clinics throughout Malaysia. Furthermore, future studies should assess the impact of pharmacist interventions on other chronic conditions, including but not limited to asthma, arthritis, cancer, Alzheimer’s disease, and dementia.

## 1 Introduction

In the past few decades, diabetes mellitus has emerged as the most widespread public health issue (1). According to global statistics from the World Health Organization (WHO), diabetes is a critical concern that impacts 415 million people globally. Additionally, its prevalence in the adult population (18 years of age and older) has increased from 4.7 to 8.5% between 1980 and 2014. Moreover, it is expected to increase further to reach an estimated 642 million by 2040 (2).

As per the National Health and Morbidity survey of the Ministry of Health Malaysia, conducted in 2018, the prevalence of self-reported diabetes mellitus in the country was 18.8% (3). Such an increased incidence greatly affects the economic burden of a country’s health care system (4). The increased prevalence of diabetes mellitus contributes to an unmanaged disease, which leads to various clinical complications. Hence, achieving optimal glycemic control is imperative to prevent both microvascular and macrovascular diabetic comorbidities (5).

Effective management of diabetes and glycemic control relies significantly on physician adherence to prescribed guidelines, and patient compliance and adherence to medication regimens play a crucial role (6). According to recent studies, medication non-compliance in patients with type 2 diabetes leads to multiple complications (7). Therefore, medication adherence, proper regimen compliance, and correct storage and usage of insulin and associated devices, are considered critical factors for the management of diabetes. Healthcare providers play a pivotal role in improving patient compliance by offering effective counseling and education about the potential consequences of uncontrolled disease (6). Worldwide, multiple studies have demonstrated the role of pharmacists in patient counseling in significantly improving clinical outcomes in diabetes mellitus. Moreover, the collaboration of endocrinologists with pharmacists in the management of diabetes has been identified as remarkable in achieving better glycemic control in patients (8–10).

Malaysia’s healthcare system is recognized as one of the best in the world. Pharmacists actively collaborate with physicians and endocrinologists in tertiary care hospitals in Malaysia through the Diabetes Medication Therapy Adherence Clinics (DMTAC).

The Malaysian Ministry of Health presented the DMTAC program to attain effective glycemic control and reduce the complications associated with diabetes in patients.

The DMTAC program requires pharmacists to collaborate with physicians to prescribe the most effective medication regimen for patients. Pharmacists are actively engaged in providing counseling to patients on diabetes mellitus, covering general management, medication frequency, and the appropriate storage and usage of insulin. In the DMTAC department, patients with inadequate glycemic control or uncontrolled diabetes are referred by the physician to a qualified pharmacist. The pharmacist conducts monthly meetings with the patient. The pharmacist is trained to counsel the patient about diabetes, diabetes management, self-monitoring of glycemic index such as; random blood glucose and fasting blood glucose, dosage regimen, adequate use and storage of insulin and insulin devices (insulin pens), details of diabetic complications, dietary and lifestyle modifications, self-management of diabetes and possible side effects of the prescribed medications. Furthermore, the pharmacist is also actively involved in monitoring and evaluating self-care and medication adherence during monthly follow-ups.

In Malaysia, various retrospective studies and single-center prospective studies focusing on DMTAC have consistently demonstrated improved medication adherence and enhanced glycemic control in patients. However, none of the studies were prospective, multicenter, or randomized controlled multicenter with 1-year follow-up. Thus, there remained a gap in determining the actual correlation between disease control, prospectively at the end of each visit and the influence of DMTAC services. The current study was carried out to observe, find out, and compare the clinical outcomes of diabetes mellitus (HBA1c, Blood pressure measurements, BMI, lipid profile, FBS, Cardiovascular incidences) with and without the active involvement of pharmacists through the DMTAC program in multicenter settings.

## 2 Materials and methods

The present study was conducted in the outpatient clinics of two different tertiary care hospitals in Kedah, Malaysia.

### 2.1 Study design

The present study was registered as a multicenter clinical trial according to WHO requirements with trial number ACTRN12621001128886 at the Australian New Zealand Clinical Trials Registry (ANZCTR).

### 2.2 Sample size

The sample size in the present study was calculated using the previous research of Butt et al. (11) to evaluate the average HbA1c in both study groups, the control and intervention groups. In total, 65 patients with type 2 diabetes from each study arm were required to detect the variations of 0.79% (8.47% versus 9.26% HBA1c) with 80% certainty (power), with a 0.05 level of alpha +SD of σ = 1.61. A value of 0.05 was the type I error probability associated with the testing of this null hypothesis. The independent *t*-test statistic would be utilized to test the null hypothesis. Moreover, another 20% dropout was added to the current study to investigate more significance in the results and the final sample size belonging to each study arm was 80 patients.

### 2.3 Procedure and randomization

Patients were selected from hospitals based on sample size calculations and with the necessary study approvals. A time window of 3–4 months was established, contingent on the patient flow in the chosen hospitals. Initially, 600 eligible patients with type 2 diabetes were included in the first list after obtaining their consent through the Informed Consent Form (ICF). Subsequently, their details were entered into Microsoft Excel, where randomization was performed to ensure an unbiased allocation of patients to the control and intervention groups. To prevent any form of selection bias in the study, a second randomization was conducted within each study group, resulting in the selection of 200 patients for both the control and intervention groups.

To prevent information contamination from the intervention group to the control group, DMTAC services were exclusively offered on two selected days per week at all participating hospitals, guaranteeing that only patients from the intervention group were present during those specific days. Additionally, all physicians managing diabetes for both study groups were well informed about the ongoing research by the Clinical Research Centre (CRC) of both hospitals, leading to the elimination of information blindness and contamination.

The study contained two groups:

#### 2.3.1 Control group

The control group consisted of adult outpatients diagnosed with type 2 diabetes who are undergoing routine management at the standard diabetes clinics of the designated study hospitals.

#### 2.3.2 Intervention group

The intervention group consisted of adult outpatients diagnosed with type 2 diabetes who are receiving regular care at diabetes clinics. In addition to standard care, these patients also underwent a pharmacist-led educational intervention at the designated study hospitals through Medication Therapy Adherence Clinics (DMTAC).

Comprehensive baseline information was documented for both study groups at each of the selected study sites.

Every 6 months, follow-up visits were carried out for both the control and intervention groups. After the baseline visits, a total of 2 follow-up visits were arranged for both study groups. At each follow-up visit, patients’ clinical outcomes and laboratory investigations were determined and recorded on validated data collection forms.

As this was an observational study, all participants’ hospital medical records were accessed, and their disease/ treatment outcome information was only collected on a validated data collection form. Only data in the form of information from the patient’s medical files was required. No other specimens were collected from patients in the form of blood, biospecimens, or any other biological samples. Educational interventions were provided to patients in the intervention group by the DMTAC pharmacists from both of the study hospitals. None of the interventions were provided by the researchers. Data were collected and stored by the principal investigator (PI).

### 2.4 Inclusion and exclusion criteria

The current study enrolled individuals who had been diagnosed with type 2 diabetes mellitus for a minimum of 5 years and had an HbA1c level greater than 8.0%. Participants receiving treatment at one of the designated study centers were included. A total of 200 diabetic patients were recruited from each selected hospital. Of these 200 individuals, 100 were assigned to the control group, while the remaining 100 were allocated to the intervention group.

However, newly diagnosed patients with type 2 diabetes, pregnant women with diabetes, diabetics with HIV/cancer, and patients with incomplete medical records were excluded from this study.

### 2.5 Outcome measures

The current study determines the impact of pharmacists on the clinical outcomes of diabetes mellitus, such as HbA1c, FBS, RBS, Lipid profile assessment, and Blood pressure measurement.

### 2.6 Statistical analysis

The data analysis was carried out using SPSS 24. The descriptive data from this study were presented as the mean with a standard deviation (SD). The normality of the data was accessed through SPSS, utilizing kurtosis + skewness testing. The data distribution was found to be normal. Afterward, independent *t*-test statistics/One-way ANOVA were utilized to determine the null hypothesis. For categorical data evaluation, the Fisher exact test or Chi-squared test was utilized for *p* value determination. However, the effect size was calculated with the help of Cramer’s V/Phi (φ).

A statistically significant *p* value was defined as *p* < 0.05.

### 2.7 Ethical considerations

Ethical approval for the study was obtained from the Clinical Research Centre (CRC) of both hospitals and the Medical Research and Ethics Committee (MREC) under the Malaysian Ministry of Health with reference number KKM/NIHSEC/ P18-1307 (13).

Patient participation in the current study was voluntary and all the participants signed the written informed consent form (ICF). Important Precautionary steps were implemented to maintain the confidentiality of the participants’ data, which was password-protected and was not shared with any participant to maintain confidentiality according to ethical standards. All data in the form of information was collected from the participants, with no biospecimen collected. There were no conflicts of interest between researchers and participants.

## 3 Results

**Figure 1:**
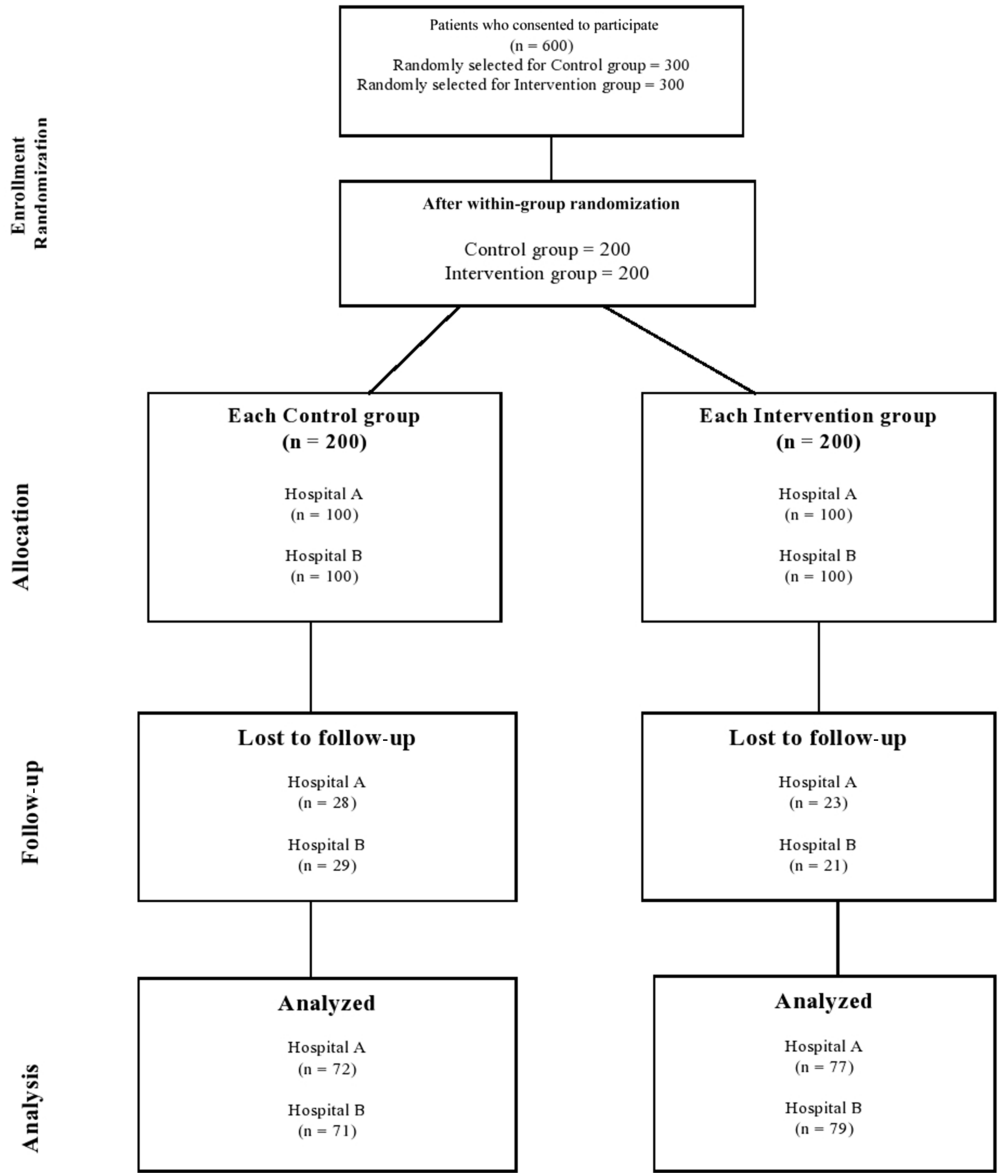
Study flow chart.

After 1 year, of the 400 patients enrolled in this study, 299 completed the two follow-up visits that were required for a total study duration of one year. Figure 1 shows the detailed sequence of patients through the study period.

A total of 25.27% (control arm: 28.5% vs. intervention arm: 22%) of the diabetic patients dropped out of the study as a result of different known and unknown reasons. Known causes included instances where certain patients in the intervention group were transferred to other Medication Therapy Adherence Clinics, such as Nephrology, Geriatric, and Respiratory units, due to complications associated with their condition. Conversely, in the control group, some patients were transferred to Medication Therapy Adherence Clinics where hospital pharmacists provided disease-related education when the desired outcomes were not achieved, making them ineligible for inclusion in this study. Additionally, some patients in the control group were transferred to different hospitals or to other locations.

Following baseline data collection, the first follow-up was conducted with a 6-month interval, and subsequently, the second follow-up took place after an additional 6 months. Consequently, the entire data collection period spanned 1 year, starting with the patient recruitment phase.

At baseline, a significant difference in the duration of diabetes was observed among the demographic variables. Moreover, a slight increase in the duration of diabetes was observed in the intervention group. No significant statistical variances (*p* > 0.05) observed among other patient demographics. In addition, no statistically significant difference was observed in the level of education between the two study arms.

The baseline demographic and clinical characteristics of the patients in the control and intervention groups are presented in Table 1.

**Table 1:** Baseline demographics and clinical characteristics of study participants.

Confounders		Frequency		Value of *p*
		CG *n*(%)	IG *n*(%)	
*Hospital name*				0.864^*^
	Hospital A	72 (48.3)	77 (51.7)	
	Hospital B	71 (47.3)	79 (52.7)	
*Sex*				0.155^*^
	Male subjects	75 (52.1)	69 (47.9)	
	Female subjects	68 (43.9)	87 (56.1)	
*Ethnicity*				0.390^*^
	Malay	106 (45.9)	125 (54.1)	
	Chinese	25 (52.1)	23 (47.9)	
	Indian	12 (60.0)	8 (40.0)	
*Age (mean, SD)*				*0.391^#^*
	–	58.61 ± 6.07 *N* = 143	61.75 ± 6.18 *N* = 156	
*Duration of diabetes (years, SD)*				***0.032*** ^***#***^
	–	9.59 ± 2.35	10.30 ± 3.24	
*Residential status*				0.438^*^
	Urban	66 (45.5)	79 (54.5)	
	Rural	77 (50.0)	77 (50.0)	
*Employment status*				0.587^*^
	Unemployed	67 (46.2)	78 (53.8)	
	Employed	76 (49.4)	78 (50.6)	
*Educational status*				0.091^*^
	No education	36 (37.5)	60 (62.5)	
	Primary education	49 (50.5)	48 (49.5)	
	Secondary education	42 (53.8)	36 (46.2)	
	College/University	16 (57.1)	12 (42.9)	
*Type of diet*				0.359^*^
	Vegetarian	80 (50.3)	79 (49.7)	
	Non-vegetarian	63 (45.0)	77 (55.0)	
*Smoking status*				0.616^*^
	Yes	27 (50.9)	26 (49.1)	
	No	116 (47.2)	130 (52.8)	
*Exercise status*				0.133^*^
	Yes	43 (55.1)	35 (44.9)	
	No	100 (45.2)	121 (54.8)	
*Type of anti-diabetic therapy*				0.520^*^
	Oral only	22 (43.1)	29 (56.9)	
	Insulin	78 (51.0)	75 (49.0)	
	Oral + insulin	43 (45.3)	52 (54.7)	

^*^Chi-squared test. ^#^One-way ANOVA test.

### 3.1 Clinical outcomes

The present study evaluated differences in glycemic index as fasting blood sugar levels (FBS) along with random blood sugar levels (RBS), glycated hemoglobin (HbA1c), systolic (BP systolic) and diastolic (BP diastolic) blood pressure, total cholesterol, high-density lipoprotein cholesterol (HDL-C), low-density lipoprotein cholesterol (LDL-C) and triglycerides.

At baseline, no significant difference was observed between the control group (CG) and the intervention group (IG). The differences in the above-mentioned parameters were recorded every 6 months and are presented in Tables 1–3 at baseline, after 6 months (follow-up 1), and after another 6 months (follow-up 2). The differences observed in the control and intervention groups are shown in Tables 2, 3.

**Table 2:** Differences in clinical outcomes between study groups at baseline (*N* = 299).

Outcome measures	Control group Mean ± SD	Intervention group	Value of p^*^
FBS (mmol/L)	14.63 ± 1.37	14.51 ± 1.47	*0.451*
RBS (mmol/L)	17.60 ± 1.14	17.86 ± 1.12	*0.056*
HbA1c (%)	11.30 ± 1.18	11.41 ± 1.10	*0.427*
BP systolic (mmHg)	137.54 ± 4.47	139.26 ± 5.39	*0.008*
BP diastolic (mmHg)	86.66 ± 5.50	87.21 ± 5.77	*0.403*
T. Cholesterol (mmol/L)	6.03 ± 0.34	6.16 ± 0.35	*0.002*
Triglyceride (mmol/L)	2.01 ± 0.18	2.06 ± 0.21	*0.026*
LDL-C (mmol/L)	2.81 ± 0.17	3.87 ± 0.29	*0.001*
HDL-C (mmol/L)	1.04 ± 0.26	1.00 ± 0.26	*0.002*

^*^Two-way ANOVA test.

**Table 3:** Clinical outcome measures at follow-up 1.

Outcome variable	Mean (SD)	Difference to baseline	95% Confidence interval		*t*-statistic (*df*)	*p*-value	Effect size (*η*^2^)
			Lower bound	Upper bound			
*FBS (mmol/L)*							
Control group	12.09 ± 1.34	−2.54	11.87	12.31	*12.51 (1, 297)*	*<0.001*	*0.34*
Intervention group	10.01 ± 1.51	−4.50	9.77	10.25			
*RBS (mmol/L)*							
Control group	15.86 ± 0.58	−1.74	15.76	15.95	*25.32 (1, 297)*	*<0.001*	*0.68*
Intervention group	12.96 ± 1.24	−4.96	12.77	13.16			
*HbA1c (%)*							
Control group	10.38 ± 1.10	−0.92	10.20	10.56	*4.82 (1, 297)*	*<0.001*	*0.07*
Intervention group	9.83 ± 0.85	−1.58	9.70	9.97			
*BP systolic (mmHg)*							
Control group	133.99 ± 3.29	−3.55	133.44	134.53	*−1.89 (1, 297)*	*0.059*	*0.01*
Intervention group	134.77 ± 3.80	−4.49	134.17	135.37			
*BP diastolic (mmHg)*							
Control group	85.15 ± 5.05	−1.51	84.31	85.99	*2.66 (1, 297)*	*0.008*	*0.02*
Intervention group	83.45 ± 5.87	−3.76	82.52	84.38			
*T. Cholesterol (mmol/L)*							
Control group	5.59 ± 0.43	−0.44	5.52	5.67	*4.04 (1, 297)*	*<0.001*	*0.05*
Intervention group	5.41 ± 0.32	−0.75	5.36	5.47			
*Triglyceride (mmol/L)*							
Control group	1.88 ± 0.18	−0.13	1.85	1.91	*−3.54 (1, 297)*	*<0.001*	*0.04*
Intervention group	1.96 ± 0.21	−0.10	1.93	2.00			
*LDL-C (mmol/L)*							
Control group	2.73 ± 0.12	−0.08	2.71	2.75	*−8.67 (1, 297)*	*<0.001*	*0.20*
Intervention group	3.01 ± 0.35	−0.86	2.95	3.06			
*HDL-C (mmol/L)*							
Control group	1.16 ± 0.03	+0.12	1.15	1.16	*7.69 (1, 297)*	*<0.001*	*0.16*
Intervention group	1.12 ± 0.05	+0.12	1.11	1.12			

*p*-value between both study groups after 6 months of baseline. Two-way ANOVA was used to find the *p*-values (*Post-hoc* analysis using the Games-Howell procedure). The *t*-statistic is further from zero than the critical values considered to reject the Null Hypothesis. C.I, confidence interval (group statistics); SD, standard deviation; df, degrees of freedom. The effect size was measured by Partial Eta Squared (*η*^2^). According to Cohen’s classification of effect size, if 0.01 ≤ *η*^2^ ≤ 0.06 = small, if 0.06 ≤ *η*^2^ ≤ 0.14 = medium, *η*^2^ ≥ 0.14 = large.

Significant improvements in clinical outcome parameters were observed at the first follow-up visit. Moreover, both groups presented statistically significant improvements in clinical outcomes. However, these improvements were comparatively more pronounced and remarkable in the interventional arm.

At follow-up 1, among all clinical parameters, the mean reduction in Random Blood Sugar was the highest and comparatively significant (*p* ≤ 0.001, *t*-statistic = 25.32).

The glycated hemoglobin (HbA1c) within the control arm was reduced to 0.92% (from bottom-line observations). Moreover, the mean reduction in glycated hemoglobin in the intervention arm was 1.58% (*p* ≤ 0.001, *t*-statistic = 4.82). A minor, but statistically notable difference (*p* = 0.008, *t*-statistic = −1.89) was observed in the systolic blood pressure (SBP) of the recruited study subjects in the control and intervention groups.

The impact of the pharmacist-influenced healthcare intervention was additionally evaluated by the statistical model, i.e., *Post-hoc* analysis using the Games-Howell method, by which phi and Cramer’s were calculated to evaluate the relationship between dependent and independent confounders. A statistically strong (*p* ≤ 0.001) association was observed between the study arms with RBS (*η*^2^ = 0.68) and a positive weak association was also observed with SBP measurement (*η*^2^ = 0.01), as shown in Table 2.

The first follow-up was followed by a second follow-up, the results of which are presented in Table 3.

At the second follow-up, there was a notable and statistically significant improvement observed in the parameters of the clinical outcome measures. Furthermore, these improvements were evident in both the control group and the intervention study arms. However, a more substantial and striking improvement was observed in the intervention study arm.

At this second visit, a significant reduction in Fasting Blood Sugar was observed, with a statistically significant mean reduction that was the largest across all outcome variables (*p* ≤ 0.001, *t*-statistic = 25.65). Additionally, in the control arm, glycated hemoglobin (HbA1c) decreased by 2.17% from the initial observations. In contrast, the intervention group exhibited a greater mean reduction in glycated hemoglobin (HbA1c) at the current time point, amounting to 3.59% (*p* ≤ 0.001, *t*-statistic = 13.79). However, the cholesterol levels of study subjects in both study arms showed minimal improvement, indicating a statistically weak positive association (*p* = 0.018, *t*-statistic = 2.38).

During each follow-up, every clinical outcome parameter was observed to be improving in both study groups. However, this improvement was statistically significant in the intervention arm with pharmacists-involved collaborative care in intervention in terms of disease and outcomes. The impact of pharmacist-collaborative care was also evaluated by post-hoc analysis using the Games-Howell method. Furthermore, the association was calculated to assess the relationship between dependent and independent confounders. A statistically strong significant (*p* ≤ 0.001) association was observed in both study arms for each outcome parameter. Another strong effect was observed with RBS (*η*^2^ = 0.68) in both groups of the present study – the parameter is presented in Table 3.

The observations at the second follow-up are presented in Table 4.

**Table 4:** Clinical outcomes measures at follow-up 2.

Outcome variable	Mean (SD)	Difference to baseline	95% Confidence interval		*t*-Statistic (*df*)	*p*-value	Effect size (*η*^2^)
			Lower bound	Upper bound			
*FBS (mmol/L)*							
Control group	11.79 ± 1.33	−2.84	11.57	12.01	*25.64 (1, 297)*	*<0.001*	*0.68*
Intervention group	7.63 ± 1.45	−6.88	7.40	7.86			
*RBS (mmol/L)*							
Control group	14.13 ± 1.34	−3.47	13.91	14.35	*12.67 (1, 297)*	*<0.001*	*0.35*
Intervention group	12.07 ± 1.45	−5.79	11.84	12.30			
*HbA1c (%)*							
Control group	9.13 ± 0.89	−2.17	8.98	9.28	*13.79 (1, 297)*	*<0.001*	*0.39*
Intervention group	7.82 ± 0.74	−3.59	7.70	7.94			
*BP systolic (mmHg)*							
Control group	135.13 ± 5.99	−2.41	134.14	136.12	*7.88 (1, 297)*	*<0.001*	*0.17*
Intervention group	129.97 ± 5.31	−9.29	129.13	130.81			
*BP diastolic (mmHg)*							
Control group	82.97 ± 5.55	−3.69	82.06	83.89	*5.60 (1, 297)*	*<0.001*	*0.09*
Intervention group	79.63 ± 4.76	−7.58	78.88	80.38			
*T. Cholesterol (mmol/L)*							
Control group	5.36 ± 0.27	−0.67	5.31	5.40	*2.37 (1, 297)*	*0.018*	*0.01*
Intervention group	5.29 ± 0.25	−0.87	5.25	5.33			
*Triglyceride (mmol/L)*							
Control group	1.76 ± 0.12	−0.25	1.74	1.78	*5.50 (1, 297)*	*<0.001*	*0.09*
Intervention group	1.69 ± 0.12	−0.37	1.67	1.70			
*LDL-C (mmol/L)*							
Control group	2.74 ± 0.13	−0.07	2.72	2.76	*4.14 (1, 297)*	*<0.001*	*0.05*
Intervention group	2.68 ± 0.11	−1.19	2.66	2.70			
*HDL-C (mmol/L)*							
Control group	1.20 ± 0.07	+0.16	1.18	1.21	*−5.70 (1, 297)*	*<0.001*	*0.09*
Intervention group	1.25 ± 0.09	+0.25	1.24	1.27			

*p*-value between both study groups after 6 months of follow up 1. Two-way ANOVA was used to find the *p*-values (*Post-hoc* analysis using the Games-Howell procedure). The *t*-statistic is further from zero than the critical values considered to reject the Null Hypothesis. C.I, confidence interval (group statistics); SD, standard deviation; df, degrees of freedom. The effect size was measured by Partial Eta Squared (*η*^2^). According to Cohen’s classification of effect size, if 0.01 ≤ *η*^2^ ≤ 0.06 = small, if 0.06 ≤ *η*^2^ ≤ 0.14 = medium, *η*^2^ ≥ 0.14 = large.

## 4 Discussion

Educational interventions led by pharmacists for lifestyle-related diseases, particularly diabetes mellitus, are well-established worldwide (11, 12). Numerous studies have shown that such interventions improve disease outcomes (11–13). Collaborative care involving healthcare professionals, especially pharmacists, consistently leads to positive disease management outcomes. In Malaysia, these services are effectively provided through Medication Adherence Therapy Clinics (DMTAC), which cover a wide range of lifestyle diseases (9). The present study is a randomized, prospective, and multicenter observational research. It constitutes one of the pioneering investigations of its kind, focusing on assessing the impact of pharmacist participation in the Medication Therapy Adherence Clinics (MTAC) program on individuals with diabetes mellitus, both with and without comorbidities. The research was conducted at two distinct hospitals located in Malaysia.

The results of the current study show that the average reduction in glycated hemoglobin (HbA1c) from the study’s initiation to its conclusion was approximately 2.18% in the control arm. In contrast, the mean reduction in HbA1c in the intervention group, where pharmacists played a role, was notably higher at 3.58%. This represents a statistically significant improvement in the intervention arm compared to the control group. The overall decrease in glycated hemoglobin (HbA1c) was observed in both the control and intervention arms of the study. This reduction can be attributed to the involvement of specialized and skilled healthcare providers in tertiary care hospitals throughout Malaysia. However, the reduction in HbA1c specifically among patients in the intervention group, was considerably greater compared to patients in the control group due to the collaborative care involving pharmacists. These results are in line with a randomized controlled study in clinical research by Lim et al. (9), according to which the decrease in average HbA1c in the intervention group was 0.91 and 0.08% in the control group, which is statistically significant (*p* ≤ 0.011) among both groups. The observations of the present study are consistent with the randomized controlled trial conducted by Butt et al. (11), which was conducted in the “Universiti Kebangsaan Malaysia Medical Centre (UKMMC) in Malaysia.” HbA1c in the study subjects was reduced significantly, from 9.677 to 8.48% (*p* ≤ 0.001) in the intervention arm of the study; however, no statistical reduction in HbA1c was observed in the control arm of the study (9.64–9.26%, *p* = 0.14). Furthermore, the findings of the current study are in line with a systematic review by Muhammad et al. (14), which explained that pharmacist collaborative care can reduce HbA1c targets up to an average of 0.75%.

The results of the present study are slightly different from those of a six-month randomized control trial, according to which the HbA1c of patients with type 2 diabetes belonging to the intervention group significantly decreased up to 0.8%. On the other hand, it was slightly higher in the control group (Jarabet et al., 2012) (15). According to a study conducted by Phumipamorn et al. (16) with a six-month intervention by pharmacists, the average reduction in HbA1c, i.e., glycated hemoglobin in the intervention study arm was approximately 0.8%. However, the HbA1c reduction in the control arm of the same study was not statistically significant. The probable reason could be the implementation of the intervention as disease education and management for the intervention group study subjects by the pharmacists. In addition to the prospective Randomized Control Trials (RCTs), retrospective research studies also presented similar results. Abdullah et al. (17) conducted a study that showed that the average decrease in HbA1c in diabetic patients was improved by 1.32% due to collaborative care by pharmacists. However, another study by You et al. (18) presented the results that the average decrease in HbA1c with the contribution of pharmacists is 1.0% with a standard deviation of ±1.7.

The study carried out by Stratton et al. (19) demonstrated that for every 1% decrease in mean HbA1c levels, there was a corresponding 21% reduction in the risk of microvascular complications. Therefore, diabetic patients with the of pharmacist-led intervention in the present study indirectly indicate that if HbA1 reduction is 3.59% in the present study, it would be indirectly associated with a 27% reduction in diabetes and approximately a 75.3% decrease in diabetic-related risks of all diabetic-related complications.

In the current study, the blood pressure of diabetic patients was also observed along with glycemic control in response to the pharmacist-led intervention group. During the baseline survey, the mean SBP of the diabetic patients belonging to the control arm of the current study was 137.54 ± 4.47. However, in the intervention arm, the mean SBP was 139.26 ± 5.38. Hence, the average mean SBP in the intervention arm of the study was comparatively higher. This might be the reason why these patients were referred to pharmacists by physicians to receive intervention. In the control arm of the current study, the average SBP was found to be around the borderline. Moreover, the average SBP in the intervention arm was comparatively higher in accordance with the blood pressure levels. Additionally, the average systolic blood pressure (SBP) in the intervention group was relatively higher, aligning with the blood pressure levels outlined in the Malaysian Clinical Practice Guidelines (CPG) for the Malaysian population. At the first follow-up, the average decrease in SBP in control arm patients was 3.55 mmHg, whereas, the mean decrease in SBP in intervention arm patients was 4.49 mmHg, i.e., statistically non-significant (*p* < 0.059, *η*^2^ 0.01). Furthermore, during the second follow-up visit, the average decrease in SBP levels in the control arm was 2.41 mmHg and in the intervention group, it was 9.29 mmHg, i.e., statistically significant (*p* < 0.001, *η*^2^ 0.17). A study by Aikens et al. revealed that each 1 mmHg increase in SBP is associated with a 2% increase in the risk of hyperglycemia (20). Therefore, the mean difference between the intervention and control groups in the current study is approximately 7 mmHg. Consequently, the mean difference between the intervention and control groups in the present study suggests that approximately a 7 mmHg reduction in systolic blood pressure could lead to a 14% decrease in the likelihood of hyperglycemia.

Along with the decrease in SBP, there was also a decrease in diastolic blood pressure, which was observed in both study groups. The average decrease in diastolic blood pressure (DBP) levels in the control arm was 1.51 mmHg. In the intervention group, this decrease was 3.76 mmHg (*p* < 0.008, *η*^2^ 0.02) during the first follow-up. However, during the second follow-up, a comparatively greater reduction was observed in the intervention group. An average decrease in diastolic blood pressure (DBP) in patients in the control arm was identified as being 3.69 mmHg (*p* < 0.001), whereas such a reduction was 7.58 mmHg (p < 0.001, *η*^2^ 0.09) in patients in the intervention arm. These observations from the present study are consistent with the findings of research conducted by Lim et al. (9), according to Lim et al. (9) the decrease in average mean SBP was 3.55 mmHg. In the control arm, it was increased up to 5.76 mmHg, which was considered uncontrolled blood pressure, according to CPG Malaysia. According to the same research (9), the average diastolic blood pressure was reduced up to 2.59 mmHg in the pharmacist collaborative care study arm. Furthermore, the results of the present study are also supported by the findings of Albsoul-Younes et al. (2011) (10). The average reduction in SBP observed by those authors was 5.50 mmHg and diastolic blood pressure was reduced up to 3.33 mmHg as a result of the intervention provided by the pharmacists. Similarly, the findings of the current study are in agreement with the work by Hammad et al. (21), which proves that the mean decrease in SBP was 4.54 mmHg, whereas the reduction in diastolic pressure was 2.21 mmHg due to the contribution of pharmacists’ collaborative care like in the current study.

The prestigious study by Adler et al. (22) proved that each 10 mmHg decrease in SBP is directly linked with an 11% reduction in the possibility of myocardial infarction in patients. Therefore, a decrease of 7.58 mmHg in average SBP in the present study’s intervention group is indirectly associated with an approximately 8.3% reduction in the risk of myocardial infarction.

At the same time, the improvement in the patients’ lipid profiles in the present study as a result of the pharmacists’ collaborative care was evident. Total Cholesterol, triglyceride, and LDL-C levels were drastically reduced. However, the laboratory values of HDL-C were significantly improved in the intervention group. Moreover, statistically non-significant differences were observed in the control arm. In this study, the average reduction in the lab values of total Cholesterol from baseline observations to the end of the study was 0.87 mmol/L, for triglyceride the mean reduction was 0.37 mmol/L, the mean reduction in LDL-C level was 1.19 mmol/L, and the HDL-C level was raised up to 0.25 mmol/Liter. These observations are highly supported by the results of research carried out by Lim et al. (9), according to which the decrease in T. Cholesterol was about 0.34 mmol/L, the mean reduction in Triglyceride levels was 0.46 mmol/liter, the reduction in LDL-C laboratory values was 0.07 mmol/liter and the laboratory values of HDL-C were raised up to 0.05 mmol/ liter, respectively, from the bottom line observations to the end of the study. Accordingly, the findings of this study are also supported by the research conducted by Jarab et al. (15) according to which the participation of a pharmacist can reduce the lab values of T. Cholesterol by 30.89 mg/dL and Triglyceride by up to 14.41 mg/dL. Similarly, the findings of this study are also in line with Wishah et al. (23), which presented that pharmacist-provided intervention can cause a reduction in the lipid profile with T. Cholesterol being reduced up to 8.40 mg/dL, Triglyceride levels being reduced up to16.9 mg/dL and LDL-C lab values being reduced up to 4.4 mg/dL. Moreover, a study conducted by Rabizadeh et al. proved that LDL-C is associated with insulin resistance and is the strongest predictor of coronary heart disease development, followed by HDL-C (24). Thus, the pharmacist intervention in the current study not only resulted in better lipid profiles in patients but also played a role in not developing insulin resistance and halting the development of coronary heart disease.

The innovative research study conducted by Grundy et al. (25) proved that the decrease in the lipid profiles of patients is directly associated to the risk reduction of cardiac diseases in patients with primary coronary heart disease. Hence, according to Grundy et al., the patients involved in pharmacist- led educational interventions are indirectly associated with a 30% risk reduction of coronary heart disease.

The current study also presented the findings of greater compliance in the intervention group patients, who were directly involved in the collaborative care with the pharmacists. After one year of follow-up, the compliance pattern was significantly better than the baseline observations through the end of the study. Diabetic patients with uncontrolled glycemic levels were transferred to insulin therapy or alternatively to dual therapy (Insulin + oral anti-diabetic agents), in accordance with the recommendations of the Malaysian CPG for type 2 diabetes mellitus (T2DM). The outcomes of the current study showed that at the follow-up visits, the majority of the patients enrolled in this study were moved toward insulin therapy or dual therapy, but this ratio was comparatively higher in the intervention arm as opposed to the control arm. These study findings are also supported by the research conducted in Malaysia by Iqbal et al. (26), which states that physicians’ adherence to the treatment guidelines in Malaysia would improve the treatment outcomes in controlling and managing the disease. Furthermore, the findings of the present study contradict the research carried out by Oude Wesselink et al. (27), which presented that there was no significant association between guideline compliance and clinical outcomes in Type 2 diabetes.

## 5 Conclusion

In general, treatment as recommended by the Clinical Practice Guidelines (CPG) for diabetes mellitus, was provided by healthcare professionals at both study hospitals and in both study groups. However, comparatively better control of diabetes mellitus with or without comorbidities could be accomplished with the involvement of pharmacist-led interventions. The outcomes of the current study provided confirmation for the effectiveness and efficacy of the DMTAC programs in Malaysia. The present study suggested that the services and responsibilities of pharmacists should be enhanced, in particular for the study subjects with uncontrolled diabetes, for the prevention of future diabetic complications.

## Data availability statement

The original contributions presented in the study are included in the article/supplementary material, further inquiries can be directed to the corresponding author.

## Ethics statement

The studies involving humans were approved by Medical Research and Ethics Committee (MREC), under the supervision of Ministry of Health Malaysia [KKM/NIHSEC/P18-1307 (13)]. The studies were conducted in accordance with the local legislation and institutional requirements. The participants provided their written informed consent to participate in this study.

## Author contributions

MZI: Conceptualization, Data curation, Formal analysis, Investigation, Writing – original draft. SA: Methodology, Software, Writing – original draft. NM: Validation, Writing – original draft. SS: Formal analysis, Methodology, Writing – original draft. RM: Writing – review & editing, Supervision, Validation. AM: Formal analysis, Methodology, Writing – original draft. AK: Supervision, Writing – original draft. MSI: Project administration, Supervision, Writing – review & editing.
